# Is AI recruiting (un)ethical? A human rights perspective on the use of AI for hiring

**DOI:** 10.1007/s43681-022-00166-4

**Published:** 2022-07-25

**Authors:** Anna Lena Hunkenschroer, Alexander Kriebitz

**Affiliations:** Chair of Business Ethics, Technical University of Munich, Arcisstr. 21, 80333 Munich, Germany

**Keywords:** AI ethics, AI recruiting, Artificial intelligence, Employee selection, Human rights

## Abstract

The use of artificial intelligence (AI) technologies in organizations’ recruiting and selection procedures has become commonplace in business practice; accordingly, research on AI recruiting has increased substantially in recent years. But, though various articles have highlighted the potential opportunities and ethical risks of AI recruiting, the topic has not been normatively assessed yet. We aim to fill this gap by providing an ethical analysis of AI recruiting from a human rights perspective. In doing so, we elaborate on human rights’ theoretical implications for corporate use of AI-driven hiring solutions. Therefore, we analyze whether AI hiring practices inherently conflict with the concepts of validity, autonomy, nondiscrimination, privacy, and transparency, which represent the main human rights relevant in this context. Concluding that these concepts are not at odds, we then use existing legal and ethical implications to determine organizations’ responsibility to enforce and realize human rights standards in the context of AI recruiting.

## Introduction

Increasingly, companies are using artificial intelligence (AI) recruiting tools to enhance the speed and efficiency of the applicant recruiting process. Especially in large companies, such as Vodafone, KPMG, BASF, or Unilever, the use of AI tools is already well-established to handle large numbers of incoming applications [1, 2]. However, AI’s application to recruitment is the subject of controversy in public and academic discourse, due to the close relation between AI-based decision-making and ethical norms and values. One line of criticism considers it problematic that important decisions affecting people’s lives are outsourced to AI, which is especially problematic if mistakes are made. One of the best-known real-world examples is the case of Amazon in 2018, where a tested AI software systematically discriminated against women in the hiring process [3]. Various researchers, therefore, have warned of the significant risk these tools’ unknown flaws, such as algorithmic bias [4], pose to organizations implementing new forms of AI in their human resources (HR) processes. Similarly, several philosophers [e.g., 5] have condemned the use of AI in recruitment, denying that AI could possess the social and empathetic skills needed in the selection process.

Still, many providers of AI recruiting tools advertise their products by claiming that they reduce bias and increase fairness in recruitment processes. In addition, widely held assumptions about the objectivity of learning algorithms contribute to a rather positive image of AI-aided recruitment among practitioners [e.g., 6, 7]. The contrast between this positive image and the ethical concerns of AI recruitment’s critics calls for a normative assessment, essential for a more nuanced view of the ethical status of AI recruitment.

This paper aims to fill this gap and provide an ethical analysis of AI recruiting to answer the question of whether AI recruiting should be considered (un)ethical from a human rights perspective, and if so, for what reason. We chose this perspective because human rights are internationally accepted as normative criterion for corporate actions and, increasingly, are integrated in soft law for business [8–10]. Human rights are overarching and comprehensive, yet also aim to be sensitive to cultural nuance [11]. Furthermore, as a legal framework, human rights carry significant implications for the moral underpinnings of business [12, 13].

The remainder of the paper is organized as follows: Sect. 2 clarifies the concept of AI recruitment; in Sect. 3, we outline the normative foundation of our approach, which is based on human rights discourse, and explore human rights’ implications for corporations and AI recruiting. In Sect. 4, which is purely analytical, we discuss whether AI inherently conflicts with the key principles: validity, human autonomy, nondiscrimination, privacy, and transparency, which represent the human rights relevant in the AI-based recruitment context. Lastly, we discuss the contingent limitations of the use of AI in hiring. Here, we use existing legal and ethical implications to discern organizations’ responsibility to enforce and realize human rights standards in the context of AI recruiting, before outlining our concluding remarks.

The contributions of our article are threefold. First, we address the need for domain-specific work in the field of AI ethics [14–16]. In examining the ethicality of AI recruiting, we go beyond general AI ethics guidelines that present overarching normative principles [e.g., 15, 17] and study in detail the ethical implications of AI usage in this specific business function. Second, our paper expands the theoretical research in the field of AI recruiting. Though various extant articles have a practical [e.g., 18], technical [e.g., 19], or empirical [e.g., 20, 21] focus, very few articles refer to ethical theories [e.g., 22] in this context (see review article [23]). To the best of our knowledge, our approach is one of the first to normatively assess whether the use of AI in the recruitment context is (un)ethical per se. By analyzing the use of AI in hiring from a human rights perspective, our paper overlaps with the work of Yam and Skorburg [11]. Nevertheless, while these authors evaluate whether various algorithmic impact assessments sufficiently address human rights to close the algorithmic accountability gap, we examine more fundamentally whether AI hiring practices inherently conflict with human rights. Third, our article provides implications for practice. By defining the ethical responsibilities of organizations, we aim to guide organizations on how to deploy AI in the recruiting process and enhance morality in hiring.

## Definition: what is AI recruiting?

We define *AI recruiting* as any organizational procedure during the recruitment and selection of job candidates that makes use of AI, whereas *AI* itself refers to “a system’s ability to interpret external data correctly, to learn from such data, and to use those learnings to achieve specific goals and tasks through flexible adaptation” [24]. This definition encompasses a diverse set of technologies, including complex machine learning (ML) approaches, natural language processing, and voice recognition.

These technologies can be applied across four commonly accepted stages of the recruiting process: outreach, screening, assessment and facilitation [25]. In the *outreach* stage, AI can be leveraged for targeted communication across online platforms and social media [26] or for de-biasing the wording of job ads to make them gender neutral and attract a diverse pool of applicants [27]. Moreover, algorithms are used to *screen* applicants’ CVs and derive a short list of the most promising candidates [19]. These screening tools are considered highly efficient, especially for top employers who receive huge numbers of applications for a single position. In the *assessment* stage, face recognition software can be used to analyze video interviews, evaluate applicants’ responses, and provide insight into certain personality traits and competencies [28]. In addition to interviews, AI-powered and gamified skill tests are used to assess further qualities, such as persistence or motivation. Therein, target variables do not need to be predefined by the company; ML algorithms can analyze the data of a company’s current top performers and determine which applicant characteristics and skills have been associated with better job performance [29]. Lastly, AI can also be leveraged to *facilitate* the selection process, for example, in scheduling activities [30].

## Normative foundation: the implications of human rights for AI recruiting

In the following section, we summarize the different implications of AI recruiting as derived from the discourse on human rights. As a starting point for our approach, we focus on human rights, given their international acceptance as a normative concept for corporate action [8–10]. To structure our review of normative approaches and discussions, we have distinguished between different coinciding discourses. These include the more general debate on business and human rights, establishing that not only states but also companies are accountable for human rights; the specific human rights implications of recruiting; and, finally, the discourse on the ethical regulation of AI. All three of these perspectives are pertinent in carving out the ethical materiality of AI usage in hiring, as they outline the responsibilities of the key actors: companies that define recruitment practices and standards and establish criteria for the judgment of AI solutions.

### Human rights and business

The discourse on business and human rights explores whether and to what extent companies must fulfill human rights responsibilities and obligations [9, 31]. Conventional wisdom suggests that business and human rights inherently stand in conflict, given the interest of companies in maximizing their profits and the intense competition they face, which enhances the pressure on decision makers to reduce costs. The notion of the primacy of profitability and fiduciary responsibility was encapsulated in Friedman’s dictum: “the business of business is to make profit” [32]. As society increasingly scrutinizes the actions of companies, contemporary theories of business ethics and corporate social responsibility have acknowledged the existence of company-specific human rights obligations [33, 34]. An emerging consensus implies that human rights are of increasing significance for business and that corporate decision makers are required to protect, respect, and remedy human rights. This notion is reflected in the UN Guiding Principles, which are grounded in the belief that business enterprises are “required to comply with all applicable laws and to respect human rights.” [35] Hence, human rights are boundaries that corporate actions must not cross, a principle that implies that certain acts, such as discrimination or violation of the human dignity of employees, are morally reprehensible.^1^ Companies are obliged to comply with these legal responsibilities “through their own activities” (United Nations General Principles, Principle 13), including business operations such as recruiting and the use of AI [36].

### Human rights and recruiting

The notion that business enterprises have to honor human rights has major implications for recruiting, which has become an important source of sustainable competitive advantage for organizations [25]. Here, the context of recruiting is dominated by diverging interests and different rights applicable between companies and potential employees. Companies have a legitimate interest in selecting and filtering out the best candidates for a certain job and also a right to information gained by checking whether an applicant fulfills the qualifications demanded by the company. This right to information is, strictly speaking, not a human right as such, but rather arises from the right to property of companies and their owners, as well as from their legal interest in an effective process that ensures the selection of the right employees. In view of HR’s relevance to an enterprise’s commercial success, the company needs to have sufficient insight into the qualities of the potential employee. Here, the limitations of collecting information and the limitations of the general right to property connect to a wider legal debate on the derogation of the right to privacy and the right to property [37, 38] as well as to the discourse on whistleblowing [39].

The rights to property and freedom of contract, however, are limited so that companies may not disregard the interests and rights of (potential) employees. The human rights perspective suggests that hiring companies have a moral duty to safeguard applicants’ rights not only in the hiring decisions they make but also in how they treat applicants during the selection process (General Act on Equal Treatment §2 [40]; [41]). The International Bill of Human Rights^2^ includes a range of rights and freedoms linked to international labor standards, such as the rights to human dignity, occupational choice, equality, privacy, education, and favorable conditions of work. In addition, the International Labor Organization’s Declaration on Fundamental Principles and Rights at Work has addressed, in particular, freedom of association and collective bargaining, forced labor, child labor, and nondiscrimination [13].

Apart from obvious implications, such as bans on child labor or forced labor, there are other major implications of human rights that have already been discussed in the specific context of hiring. Among others, Alder and Gilbert [41] refer to the right to personal dignity. The applicants’ right to dignity requires that care be taken when it comes to potentially invasive assessment techniques such as personality tests and drug testing. The US Employee Polygraph Protection Act (EPPA) forbids private employers from using most lie detector tests, considered disrespectful and demeaning. Similarly, managers have a duty to preserve individuals’ right to privacy by safeguarding their personal information and exercising discretion when conducting background checks [41]. The right to privacy suggests that applicants have also the right to deny statements or withhold information on such topics as marriage, pregnancy, or religious affiliation, all of which could potentially be used for purposes of discrimination. Some legislation even stipulates the notion of a right to lie. The right to privacy is closely connected with anti-discrimination regulations, which derive primarily from the right to equality (one of the earliest constitutionally guaranteed rights) and are mandatory for companies (General Act on Equal Treatment §12 [40]). These regulations protect applicants’ right not to be rejected on the basis of a non-work-related characteristic such as age, gender, or ethnicity. Given the power asymmetry between applicant and employee, some scholars have expressed the view that applicants also have a right to be told the truth. Alder and Gilbert [41] have argued that managers have a moral duty to be upfront with applicants, providing them with honest assessments, updates of their status in the hiring process, and realistic previews of the job. Finally, all these rights link up to the key norms on which market economies are based, namely, the general right to freedom and the specific right to occupation that are necessary for realizing and expanding human autonomy.

In a nutshell, the pre-existing discourse on human rights in recruitment entails key normative implications for the management of the recruiting process. The first implication is that the process of hiring and access to jobs are highly relevant for many human rights in that these connect the larger debates on freedom of occupation, transparency, and human dignity with labor law and nondiscrimination legislation. The second implication is that anti-discrimination and privacy norms are closely linked and support each other in realizing dignity in the workplace. Therefore, the human rights perspective on AI recruiting has to be aware of these important connections.

### Human rights and AI

The existing literature has examined, apart from the broader implications of human rights for enterprises and recruiting, the more specific implications of human rights on the use and development of AI. The starting point of the debate on these latter implications has been that the properties of AI solutions make this technology unique, differentiating it from older technologies such as computers, airplanes, or nuclear power plants. These properties, such as automated decision-making, use of historic data, access to private data [42, 43], and AI’s black-box character [44, 45], highlight potential areas of human rights violations, as they could stand in a more general—perhaps even inherent—conflict with specific human rights, as discussed in the literature [36].

These looming conflicts AI may have with a series of human rights and other normative principles (such as happiness or economic growth) have given rise to an intense debate on the regulation of AI. Several ethics guidelines, including the Montreal Declaration for Responsible AI [46] and AI4People’s principles for AI ethics [17]^3^ have been released by various stakeholder groups.

### Human rights and their implications for AI recruiting

By combining the human rights requirements for recruitment with the discourse on AI ethics that addresses the critical properties of AI, we can derive the specific human rights implications of AI recruiting. These implications depict the analytical tool for our ethical examination, which addresses the following aspects:

- *Validity* AI is developed by human beings, who are not always perfect in their judgment and who will make mistakes in designing, programming, and using AI solutions. These mistakes might result in human rights violations, for example, when it comes to injuries or psychological stress incurred by ill-calibrated AI solutions (compare with Floridi et al.’s [17] principle of non-maleficence). However, the validity of AI recruiting can be considered a precondition for its ethicality, given companies’ need to find the right candidate. Along with efficiency, the validity of the data-driven predictions made by AI serves as the main determinant for judging the superiority [or beneficence] of AI solutions over traditional recruitment practices. This connects to the larger debate on how AI can promote human rights.
- *Autonomy* AI might reduce human involvement, as human beings cede certain decision-making or analytical tasks to automated machines. As a result, certain applications of AI could conflict with the right to human self-determination and threaten human freedom if they render certain choices obsolete. Therefore, AI recruiting tools should only be used to the extent that they do not limit human autonomy, so as not to conflict with human dignity and the right to occupation.
- *Nondiscrimination* Data sets are susceptible to many types of bias [51], increasing the likelihood that AI that is reliant on historic data will fail in realizing its aims. If a decision made by AI impacts human beings, especially in the selection of job candidates, AI might lead to discrimination. However, the right to equality provides the basis for countering this vulnerability at all costs and makes nondiscrimination a prerequisite for the use of AI in recruiting. Notably, nondiscrimination and validity might not be the same in recruiting, as there might be specific legal obligations to respect certain quotas or to respect the rights of disabled persons (see Sec. 2, U.S. Rehabilitation Act of 1973).
- *Privacy* AI decisions are typically based on a specific data input. The input used by an AI solution could conflict with the human right to privacy if the data was obtained by violating ethical principles (e.g., without the applicant having consented to its use). This risk is magnified by AI’s ability to access applicants’ personal information using, for example, facial recognition software. As addressed by regulations in the traditional context of recruiting (Sec. 2 U.S. Rehabilitation Act of 1973), data privacy is another important ethical concern in AI recruiting.
- *Transparency* The outcomes of AI decisions are beyond the full control of human beings, making it difficult to trace responsibilities [17]. Literature on AI has referred to this aspect as its black-box character, as it is difficult for its users to understand why the algorithm has decided in a certain way. However, the right to be told the truth and the right to lodge a complaint when applicants feel treated unfairly (General Act on Equal Treatment §13 [40]) make it necessary for AI recruiting to be transparent.

Table 1 summarizes the implications for AI recruiting related to the underlying human rights and AI properties. However, these implications require a more detailed examination, explicitly for understanding the specific conditions for AI use in the recruiting context.

**Table 1:** Underlying human rights in recruiting, AI properties, and implications for AI recruiting

(Main) underlying human rights in recruiting	AI properties	Implications for AI recruiting
Right to property	Data-driven predictions Programming by humans	*Precondition:* Validity
Right to freedom (and of occupation)	Automated decision-making	Autonomy
Right to equality	Use of historic data Risk of algorithmic bias	Nondiscrimination
Right to human dignity	Access to and storage of private data (e.g., through face recognition)	Privacy
Right to be told the truth	Black-box character (AI is hard to explain)	Transparency

## Ethical analysis: is AI recruiting unethical per se?

In the following, we explore the question of whether AI recruiting should be considered unethical per se. We distinguish between actions that inherently—and thus per se—conflict with human rights and actions that present a contingent conflict with human rights [see 36]. Individuals’ and organizations’ actions conflict inherently with human rights if they constitute a violation of human rights irrespective of circumstance. Based on our theoretical discussion in Sect. 3, we opt for human rights as our concept for companies’ ethical actions. Moreover, we integrate utilitarian and other approaches to ethics if they are helpful for interpreting human rights or if our analysis touches areas where human rights implications or established legal conventions do not offer straightforward solutions [34, 52]. The remainder of Sect. 4 is structured as follows:

In the first part (Sect. 4.1), we examine whether AI recruiting fulfills the precondition of providing a valid assessment of applicants. We consider this to be a necessary prerequisite because utilitarian theories of effective altruism [53] argue that ethicality involves the criterion of improvement of outcomes: status quo post must surpass status quo ante. Thus, unless AI recruiting is superior to traditional recruiting, using this technology is not only unethical but also possibly inefficient. In the following Sects. 4.2–4.5, we discuss ethical issues beyond validity, including human autonomy, nondiscrimination, privacy, and transparency. In assessing each of these principles, we address the potential reproaches against AI recruiting as well as the counterarguments for each. Table 2 summarizes this section’s discussion and the implications for organizations, which will be outlined in Sect. 5.

**Table 2:** Summary of ethical analysis of AI recruiting and implications for organizations

Ethical principles	Is AI recruiting inherently unethical?	Implications for organizations
*Precondition* Validity	*Reproach* Lack of empathy and social intelligence Missing scientific validation *Counterargument* Validity of decisions depends on what activity AI is used for Data-driven predictions are better than human ones	Establishing mechanisms for auditing and quality control Ensuring statistical expertise in HR departments Using AI for objectively measurable requirements Using AI as complementary recruiting tool
Autonomy	*Reproach* Dependence on AI-made decisions Reduction of chance to perform for applicants Dehumanization of hiring process Lack of control of every single step by recruiters *Counterargument* Applicants always depend on others’ decisions Humans are not inherently better interview partners than AI AI allows recruiters to have control over final decisions	Using AI as additional recruiting tool Establishing human oversight over process Creating transparency/ explainability reports
Nondiscrimination	*Reproach* Risk of algorithmic bias Risk of standardized discrimination Unfair treatment of nonstandard/disabled people *Counterargument* AI is never inherently racist but may be thus programmed/trained by humans AI may reduce human bias Reconfiguring AI to prevent bias against disabled people can offer a chance for inclusion	Auditing AI with regard to bias and discrimination Validating AI tools for nonstandard people Implementing diverse data scientist teams
Privacy	*Reproach* Access to additional types of data (e.g., sexual orientation) Collection and usage of many data points *Counterargument:* Firms can define and control the input data used and stored	Obtaining consent for data use from applicants Establishing data minimization: collection and storage of minimal and relevant data
Transparency	*Reproach* Black-box character: lack of transparency for the single case *Counterargument* Transparency for the general mechanism is given (e.g., in the form of open code) AI may enable regular updates and timely feedback for applicants	Disclosing selection and success criteria Reducing complexity of algorithms Creating transparency/explainability reports Communicating about discrimination cases and number of claims

### Precondition: is AI a valid tool in the recruiting and selection process?

Considering that many companies have already implemented AI technologies in their recruiting process, we assume that AI recruiting is time and cost efficient, something research agrees on [26, e.g., 54–56]. However, critics warn about AI recruiting’s potential constraints in terms of validity. One such argument states that AI represents only a simplified model of human behavior that is restricted to a set of measurable behavioral dimensions [4, 57, 58]. Thus, AI lacks empathy and cannot detect applicants’ emotional intelligence, which reduces the validity of an AI assessment [5]. Although AI may be able to recognize and imitate emotions with sensors (known as *affective computing*), it cannot understand complex emotions and feelings. Complex forms of sadness, such as self-pity, regret, and loneliness, are just as unreadable as complex forms of joy, such as schadenfreude, pride, and confidence. AI also cannot perceive and understand values or charisma. The same applies to many contexts where psychometric quantifications are inherently incapable of capturing contextual meanings of competence. One can try to program values into AI—but nuances will be lost [59–61]. Therefore, AI cannot assess an applicant’s personal or team fit or determine whether an applicant is truly motivated or reflective—or whether their statements are substantiated.

From our point of view, however, this argument against AI recruitment tools can be weakened by the fact that team fit and social intelligence are only two criteria among many in the recruiting process. Even in non-AI-based procedures, the screening and shortlisting of CVs is based on fixed and quantified criteria, such as average academic grades or months of prior job experience. These sort of criteria could be easily managed by AI. This example also leads to the question of whether academic grades are an effective predictor of subsequent performance at all and highlights the added value of another feature of AI: based on ML and the data of current top performers, AI can assess which characteristics make an applicant a good fit for a given role, thus enhancing the selection process’s accuracy [18, 62].

Again, it can be argued that AI tools are often not scientifically validated but have emerged as technological innovations only. Similarly, the underlying criteria for the prediction of job performance may not be derived from scientific research programs [63, 64]. Moreover, ML algorithms predict future human behavior based on historical data, ignoring novel patterns and parameters [65]. Therefore, predictions are often proven wrong because of changes in the overarching ecosystem [66, 67]. However, we think that it is questionable whether people, with their subjective perceptions and assessments, perform better than AI in this regard. Because AI is data-based and can process a much larger range of behavioral signals than humans can, AI may even outperform human inferences about future performance in accuracy and validity [18, 68]. This is also in line with Kahnemann’s [69] findings that algorithmic predictions generally perform better than human ones and suggests that whenever we can replace human judgments with formulas, we should at least consider it.

Overall, we think that the use of AI could contribute to more efficient and more valid recruiting decisions. Although AI alone cannot capture all potential job criteria, it is not a non-valid tool per se. Consequently, AI decisions’ validity depends on the activity for which AI is used for. Assigning appropriate tasks to AI therefore requires recognition of its shortcomings, e.g., its reductionist nature that cannot interpret contexts. That being said, validity is a contingent rather than inherent limitation to AI development and deployment in a hiring context.

### Does autonomy inherently conflict with AI recruiting?

Autonomy has been classically seen as expression of the right to freedom and self-determination in combination with more specific rights, such as freedom of occupation and freedom of movement. Although autonomy’s importance has been emphasized by various scholars [e.g., 70, 71] and in various frameworks [14, 17], its exact meaning remains disputed. Relevant questions for the interpretation of autonomy are as follows: What degree of human control is implied by the concept of autonomy? Should we try to realize human control in areas that have not yet been controlled? Autonomy’s implications depend on the answers to these questions. One might argue that human actions should not be constrained by technologies—compared to the ex ante status quo—and that humans should have control over the outcome. Here, we often encounter the notion of meta-autonomy, defined as the voluntary decision to “delegate specific decisions to machines” [17]. Other positions argue that human actions should be enhanced through technologies and that limits should be imposed on technologies [72].

In the context of AI recruiting, AI generates implications for the autonomy of not only the applicants but also the recruiters. Hence, in our analysis, we embrace both of these perspectives. Considering the *applicant perspective*, first, one may argue that the use of AI tools conflicts with applicants’ autonomy. By interacting with an AI instead of humans, applicants lose the opportunity to get to know the company in the form of future colleagues and to evaluate whether the company culture fits their needs and expectations, fully depending on the AI-made decision. Thereby, the asymmetry of time and effort investment increases: applicants invest the same amounts of time and effort as required for human-based procedures, whereas companies automate the process, saving time and money. However, regardless of the recruiting procedure used, applicants are always subject to the company’s process and depend on others’ decisions. Thus, in this regard, we do not see any impact on applicants’ autonomy. Without any personal interaction in the process, it may be even easier for applicants to accept rejection and reorient themselves afterward.

Second, one may argue that candidates’ autonomy is reduced because they cannot demonstrate all their empathetic, social, and soft skills in interviews with AI because the latter cannot fully value them. In this way, AI interviews may even lead to changes in applicants’ behavior, such as using special buzzwords that the AI will recognize. However, we would counter that human interviewers are not always better listeners or conversation partners in interviews. In fact, applicants may feel less embarrassed when sharing personal experiences with an AI than when doing so with a human. Moreover, adapting one’s behavior to an interview partner applies to not only AI interviews but also face-to-face (FTF) interviews with different types of interviewers.

Lastly, a frequent line of argument is that AI recruiting represents a conflict with human autonomy because weighty decisions are taken over by AI with huge impact on human lives. This stands in direct conflict with the meaning of human rights because it leads to a dehumanization of the recruiting process and a devaluation of human lives, especially when these tools are used for only certain types of jobs and applicants (e.g., low-impact jobs and not top-manager positions). Furthermore, although recruiting can become more efficient by using AI tools, it can ultimately lead to mechanizing the hiring process, leading to little or no direct human contact between individual applicants and the future employer [4]. This might lead to the reification of interpersonal relationships, whereby both applicants and recruiters would experience a loss of individuality and autonomy [4, 73, 74].

When taking the *recruiters’ perspective* to analyze whether AI recruiting conflicts with autonomy, we must consider the differing interpretations of autonomy and their underlying expectations regarding human control. If autonomy is understood as the control of every single step in the recruiting process, AI recruiting may indeed conflict with this concept. When AI applications take over certain activities, including data analyses and decision-making, or at least shape human decisions by interfering with deliberation processes, this results in meta-autonomy and a reduction of control for recruiters [75]. The more recruiters’ decision-making is substituted by AI, the less opportunities and autonomy recruiters will have to make their own decisions, whereby their learning capacities will be reduced [4]. This reduction of control and autonomy for recruiters may be particularly problematic if competitive pressure forces companies to use AI. Therefore, companies might opt for cost-efficient solutions at the expense of quality standards. This applies specifically to scenarios in which recruiters must process large volumes of applicants under time pressure. However, the assessment differs when understanding autonomy in the sense of end control. End control is provided to recruiters when they can overrule AI decisions or when AI is used as an additional recommendation tool, but human recruiters make the final decision about who is offered a position. Thereby, realizing human autonomy may depend on whether the team of recruiters understands the rationale of the AI solution and decision. In this case, AI recruiting would not be unethical per se, but it would require that the criteria and algorithms behind each hiring decision be explainable and known by the company. Likewise, recruiters would have to consider additional mechanisms for quality assurance. For example, randomly selected applicants who are eliminated during the AI-based process could be reevaluated by a human evaluator as a check.

Although we acknowledge that AI use may lead to a dehumanization of the recruiting process, AI usage in recruiting does not constitute an inherent breach of human rights according to our understanding. A specific debate concerns the notion of statistical dehumanization that reduces human beings to a number [76]. Similar views have been raised in the press, arguing that large numbers entail a dehumanization tendency. In our view, however, this is an ethical point that is excessively fundamental. Even today, companies are confronted with high numbers of applications that make it difficult to concentrate on individuals. One way out might lie in the aforementioned idea of allowing for exemptions from AI hiring solutions through a random review of individual cases to avoid a systematic dehumanization. Nevertheless, we consider the dehumanization argument to be a philosophical question that, first, is generally directed against any technological progress that reduces human interaction and, second, leads to further philosophical questions, such as the following: Which measures should society employ to regain humanity? Because this question is too fundamental in nature to be solved within our contribution, we treat it as an underlying assumption behind contemporary recruiting practice. Therefore, the perspective of AI solutions as conflicting inherently with human rights originates in a specific interpretation of human oversight.

### Does nondiscrimination inherently conflict with AI recruiting?

The right to nondiscrimination derives primarily from the right to equality. However, it has only recently been applied in private law. Beyond the controversial debate on quotas, diversity, and specific interpretations of the right to equality, we maintain an understanding of nondiscrimination meaning that everyone should have the same chances, regardless of personal attributes, such as ethnic, cultural, and migration backgrounds and gender. The mathematical term for nondiscrimination is that there is the same likelihood for a specific outcome given the same properties of the individuals being assessed (compare: Basic Law of the Federal Republic of Germany, Art. 3). Although discrimination entails different dimensions that transcend mathematical formulations [77], the presented approach presents a key threshold of the mathematical process underlying AI hiring.

Do AI recruiting tools per se discriminate against certain groups of applicants? The Amazon case illustrates that the use of AI in recruiting may introduce algorithmic bias due to poorly trained algorithms [e.g., 58, 78], which may result in (unintended) discrimination against certain applicant groups [e.g., 51]. Critics argue that such discrimination by a machine is even worse than discrimination by a human being because algorithmic bias standardizes and magnifies discrimination, which could also result in institutionalized racism [26, 29]. Moreover, AI may introduce new types of biases, which are not yet defined within nondiscrimination literature [79]. However, in many contexts, it is not feasible to formalize all dimensions and context-dependencies of discrimination in such a way that the extent of AI discrimination can be compared to that of human discrimination. This is also true, for example, when it comes to intersectional discrimination.^4^

However, we would argue that AI is not inherently racist and merely follows codes and criteria that are programmed by humans. Thus, the original source of algorithmic bias is human—either in the form of human behavior that the AI simulates or in the form of a programmer who (deliberately or unintentionally) programmed the AI in a racist manner. Nevertheless, we admit that adverse effects can occur when AI is used for recruiting, bearing an ethical risk. Here, the question arises of whether the risk for such algorithmic bias should be considered unethical. Although algorithmic bias may be much easier to detect and remove compared with human biases [7, 56], a conflict between AI recruiting and nondiscrimination may emerge if one argues that the pure risk of discrimination delegitimizes the use of AI.

However, it can be argued at this point that even today’s human-based selection procedures are not free of bias. Rather, the opposite is the case; scientists broadly agree that the practices currently in place are far from being effective and unbiased [e.g., 7, 81] and that AI has the potential to reduce human bias in these processes. For example, AI can address bias in the form of gendered language in job descriptions, making them gender-neutral and more inclusive [82]. Moreover, in the screening and assessment stages, subjectivity can be reduced by using algorithms that evaluate all applicants against the same criteria, thereby reducing human bias related to applicants’ physical appearance because AI can be taught to ignore people’s protected personal attributes and focus only on specific skills [83, 84]. Thus, if one argues that AI should be considered ethical as long as it has the potential to reduce human bias, we do not see an inherent conflict between human rights and AI recruiting.

Another line of argument states that the standardized process that comes along with AI recruiting triggers an unfair treatment for nonstandard applicants, such as disabled people. Scott-Parker [85] argued that when considering disabled people, fairness does not mean making the recruiting process more consistent and standardized, but rather making the process more flexible to generate equal opportunities for all applicants. This flexibility is not provided by highly automated and rigid AI recruiting processes, which are not yet validated for disabled people and ignore the impact of disabilities on voice, word choice, and movements, among other factors. For example, gamified assessments are often difficult for people with only one hand, in wheelchairs, or who are color-blind, thus discriminating against disabled people. Scott-Parker [85] called this “disability bias,” which is crucial in the AI recruiting context but is not yet often referenced in the AI debate.

We fully support this reasoning and concern; however, we do not consider it to fundamentally conflict with AI recruiting. Instead, it underscores the following needs: for AI recruiting to be validated for disabled people, to include disabled people in original databases, and to generate equal chances for all applicants. We would go even further, arguing that reconfiguring AI to disabled persons’ needs could even be a chance for inclusion.

Overall, we argue that AI recruiting does not inherently conflict with the principle of nondiscrimination, but potential systemized, algorithmic bias constitutes a contingent limitation. Although algorithmic bias may occur unintentionally and be based on unknown criteria, we consider this rather a problem of the AI tool’s validity, which should be correctly trained and programmed to work in the same way for all groups of applicants. Thus, technical due diligence and auditing regarding valid data sets and algorithmic designs are crucial to keep the risk of algorithmic bias low.

### Does privacy inherently conflict with AI recruiting?

One the one hand, privacy can be considered an essential part of human dignity and, thus, an intrinsic human right. Likewise, privacy can be derived from Articles 12, 18, and 19 of the Universal Declaration of Human Rights [86]. This understanding has been promoted, for example, by the German Federal Constitutional Court, which has interpreted a person's intimate sphere as a central human right. Thus, the court stated that the right of personality belongs to the essence of human dignity [87]. Thus, this right enjoys special protection against encroachment by others for commercial or artistic purposes. On the other hand, the right to privacy can be derived from the idea that individuals have the right to conceal information from others. Therefore, it might be considered an instrumental right because it allows individuals to engage in activities or to have preferences that are not shared by everyone or that are scrutinized by societies. Throughout history, sexual minorities have been often targeted by social stigma, which is ongoing. To the same extent, information concerning people’s ethnic backgrounds has been used to commit human rights violations.

On the contrary, utilitarian approaches would challenge privacy’s innate value. These would argue that personal privacy must be balanced with other aims, such as economic efficiency or societal safety and health (as contemporarily discussed in the context of action against COVID-19). The key question, therefore, is as follows: What type and amount of data is a potential employer allowed to collect and store concerning applicants? With the development of the General Data Protection Regulation (GDPR), privacy is already a regulated area in hiring. This regulation is aimed to protect EU citizens’ rights by governing how to collect, store, and process personal data. Moreover, individuals have the right to conceal from employers any personal information that is irrelevant for the fulfillment of the potential job task (e.g., sexual orientation).

Does privacy inherently conflict with AI recruiting? To answer this question with “no,” the GDPR states that applicants in a recruiting process must have the opportunity to explicitly consent to the use of their data. However, an ethical dilemma emerges at this point because of the power asymmetry in the job market between employers and applicants. This means that generally applicants may be unable to refuse the use of certain personal data without being disadvantaged in the process. However, this dilemma is not caused by the use of AI, but applies to the general context of hiring as well as to human-led processes [88]. The same is true for the argument that it is unethical to collect social media data for hiring purposes when users generally use social media platforms for other purposes [29, 64]. It is questionable whether social media is a good information source or a reliable indicator of job performance [19]. However, this discussion on the use of social media information in the hiring context is not new. A study in Sweden showed that at least half of the interviewed recruiters scanned candidates’ social media profiles at some point before hiring [81].

Some of AI recruiting’s inherent properties distinguish it from traditional recruiting practices, and we will focus on whether these properties conflict with the right to privacy. First, AI recruiting allows for *access to more types of data than human recruiting*. For example, AI in the form of face recognition tools and prediction algorithms may forecast which candidates are most likely to become pregnant or reveal candidates’ sexual orientations [22, 89]. This access to candidates’ personal attributes conflicts with their privacy rights and increases the risk of information misuse and discrimination [83]. Through AI use, applicants are facing increasingly invasive methods of information gathering, which are expanding from applicants’ work life to social and even physiological domains [4].

Second, an inherent property of AI recruiting is that generally, this approach involves *the collection and use of more data for decision-making than human recruiting*. Whereas a human assessment is mainly based on an interviewer’s intuition and value assessment [81], an AI tool automatically captures millions of data points from applicants’ behavior, such as their verbal and body language, for a data-driven assessment of personality [90]. On the one hand, this may lead to a more data-driven and objective assessment of applicants; on the other hand, one could argue that this increased amount of collected and stored data may conflict with applicants’ privacy rights.

However, from our perspective, these two properties of AI recruiting do not inherently conflict with the right to privacy. Although AI enables organizations to collect more data and access additional types of data, it still depends on the organization to determine and define which kinds of data the AI should collect, store, and use as input for the selection process. As long as the data collected refers to candidates’ personality traits or skills that are relevant to the job, we would not consider the use of additional data as inherently unethical, acknowledging that the distinction between relevant and irrelevant information can be blurred sometimes. However, individuals with a strong focus on data privacy might have objections to this view and consider the collection and use of certain data, such as biometric data, to be an inherent limitation of AI-based hiring.

### Does transparency inherently conflict with AI recruiting?

Transparency, which typically goes along with interpretability and explainability, has been widely discussed in AI ethics literature [1]. However, most sources of a right to transparency are not of constitutional origin but rather are derived from ordinary law. For example, the GDPR warrants a “right to explanation,” by which people can ask for explanations about (algorithmic) decisions made about them [91]. Similarly, some scholars have assigned applicants the right to be told the truth [41], whereas others have philosophically argued that there is a fundamental, moral right to ex post explanations of algorithmic decisions [92]. Individuals must understand our society’s functioning and be able to develop the right strategy to apply for jobs. In the AI recruiting context, knowing the rules of the game provides applicants the assurance that they are treated fairly.

Regarding transparency, the key question concerns the extent to which developers must disclose details on algorithms [93]. Here, the literature is divided, and there have been concerns regarding whether it is possible to establish full transparency in AI. Does the right to transparency mean generating an understanding of how the algorithm generally operates (e.g., how the algorithm uses data and weighs specific criteria)? Or, does transparency also imply disclosing the conditions and explanations for each individual algorithmic decision?

Given technology’s current state, AI does not always meet this latter requirement because complex algorithms learn from millions of data points and can become too complex to be fully understood by even those programming them. Thus, it can become difficult to explain in detail what factors drive particular decisions, giving AI a black-box character [63]. In the recruiting context, this limitation of AI is ethically critical because the decisions made by AI are highly relevant to people’s lives and because insufficient explainability bears the risk of obscuring discrimination [e.g., 29]. In the event of hiring decisions made without recruiters exactly knowing why and how the AI generated decisions, applicants could perceive the decisions as arbitrary or nonsensical, resulting in complaints of unfairness, feelings of frustration, or disengagement [4]. However, if transparency is understood as regarding the general mechanism behind an AI tool, AI can meet this requirement. The general code is determined by the programmers, who create and adapt it for their needs and accordingly have a complete understanding of it. Often, general AI algorithms are openly accessible.

We would argue that the required level of transparency in recruiting lies between the two mentioned levels. Because hiring decisions highly impact people’s lives, it should be comprehensible which data is used by the AI—which should also align with the right to privacy—and which criteria are used to evaluate candidates. Moreover, to hold AI-enabled decision-making systems accountable for their outcomes requires more than knowing their code; rather, one must clearly understand how the system works and be able to reconstruct the ex post reasons behind the AI decisions [94]. However, we do not believe that every single AI decision must be explainable down to the last detail; this is not expected of human decisions either. For example, even an interviewer cannot explain in detail or in a quantifiable way why a candidate is likeable to them or why they think that a candidate would be a good fit for the team. AI recruiting can even constitute an opportunity for greater transparency in the form of regular updates and timely feedback for applicants throughout the recruiting process. Chatbots may inform candidates about progress during the process, and AI technology may be used to generate applicants’ preliminary personality profiles, which would provide them data-driven insights on their strengths and areas in need of development [64].

Overall, we would not consider AI recruiting to be inherently unethical from a transparency perspective. However, a trade-off between an algorithm’s accuracy and explainability may emerge, assuming that increasing algorithms’ complexity increases their accuracy. Thus, we consider it a necessary condition that organizations understand and can explain how AI operates and what data and criteria are used for AI-based decision-making. This constitutes a technical challenge and a contingent limit: building AI tools that produce explainable results.

## Implications for and responsibilities of organizations

Our ethical analysis illustrates that the specific properties of AI recruiting might conflict with human rights. However, they constitute contingent rather than inherent limitations, unless one adopts an interpretation of the mentioned principles, such as human autonomy, data privacy, and transparency, that is highly restrictive. Therefore, we conclude that AI recruiting should not be considered inherently unethical. We argue that the risks related to AI recruiting are not inevitable consequences of using AI in recruiting and that they instead arise from inflated expectations and can be exacerbated by unreflective use of AI recruiting tools. Therefore, an ethical implementation of AI recruiting comes with far-reaching and challenging implications for organizations. Their responsibilities derive from legislation series of normative sources, such as the UN Guiding Principles on Business and Human Rights [35] and other human rights codifications. Here, one quintessential aspect is proactive engagement with societal stakeholders—for example, job candidates, as key stakeholders in this context—to achieve broad feedback on AI hiring’s ramifications [95, 96]. Moreover, the normative principles derived from human rights codifications and discussed in this paper generate a set of general implications for AI governance (e.g., [17]) and specific implications for organizations seeking to implement AI recruiting.

*Validity* We understand the validity and quality of AI tools as a fundamental condition for ethical use. Only tools that work as they should can guarantee fair treatment of applicants. To reduce any error-proneness (e.g., algorithmic bias), companies must utilize auditing instruments and mechanisms for quality control. Such monitoring requires adequate levels of data and statistical skills, along with enhanced statistical expertise in HR departments, within the companies using these AI tools. Furthermore, companies must ensure that AI tools are used only for activities they can accurately perform. According to the current state of technological progress, AI tools are suitable for evaluating objectively measurable characteristics of applicants, including specific skills measured by gamified assessment tools. However, criteria such as social skills and team fit should continue to be assessed by humans as long as there are no valid and scientifically tested AI-supported tools for this purpose. Overall, AI should be seen as a complementary tool in the recruiting process, supporting recruiters with data-driven analyses and predictions and thus enriching the process; it should not be seen as a complete substitution for human-led recruiting tools. Hiring decisions should always be made by an AI-informed human rather than by AI alone.

*Autonomy* This same aspect—using AI as an augmentation rather than a sole recruiting tool—also addresses an implication of the ethical principle of autonomy for AI recruiting. AI should not fully substitute humans in the recruiting process because the personal interaction between recruiters and applicants is important to counteract the process’s dehumanization. Companies should demonstrate to applicants that they are valued and perceived as individuals by providing them the chance to get to know the company and promoting their autonomy. Furthermore, human supervision of recruiting decisions should be established to ensure that companies maintain human control over final hiring decisions and enable recruiters to correct or adjust AI-provided decisions or recommendations. As discussed above, this control will require that recruiters understand the rationale of AI solutions and decisions to ensure that hiring decisions are explainable (e.g., in the form of transparency reports).

*Nondiscrimination* Because all ML algorithms use historic data, the risk of algorithmic bias due to biased data sets emerges, which may endanger the ethical principle of nondiscrimination. Thus, a dedicated auditing of AI software and its underlying databases, focused on bias and unintentional discrimination, is required in the recruiting context. Even if some AI-made predictions and decisions are not exactly traceable in individual cases, companies must ensure—and here, they bear the burden of proof—that they are not discriminatory. To achieve this, different approaches are available: Some AI software vendors delete any information that can unconsciously predict a candidate’s gender to circumvent unconscious bias. An alternative approach is to proactively collect social category data and then ensure that they are not used as evaluation criteria to eliminate any risk of discrimination. Moreover, open-source tools can facilitate systematic bias checks. As discussed above, this claim of nondiscrimination also applies to nonstandard and disabled candidates, for whom AI tools must be equally validated to generate equal chances for all applicants. In this context, the use of diverse data scientist teams who are aware of this risk and check for implicit assumptions may serve as a concrete measure to prevent the creation of discriminatory codes and foster inclusion and equity in AI.

*Privacy* As in regular recruiting processes, AI recruiting companies should obtain applicants’ consent to data use and carefully protect all sensitive data. In doing so, companies should not leverage their position of power and instead collect and use only data relevant to the hiring decision (i.e., data relevant to assessing whether an applicant is suitable for the job), following the general principle of data minimization. This principle must also apply when AI is used for data capture—for example, in the form of face recognition software—even if the form of AI in question could predict private or sensitive candidate information, such as migration background or sexual preferences.

*Transparency* ML algorithms are learning from millions of data points and deriving recommendations that are hard to explain, even for the programmers who create them. Nevertheless, we argue that organizations must provide a certain level of transparency regarding the algorithmic techniques and data sets they use and regarding the drivers behind individual decisions, making the conditions for AI recruiting challenging. As discussed above, it is a company’s responsibility to disclose the general selection and success criteria of applicants in their processes, even if they may not be able to explain every decision in detail. This responsibility may even require companies to reduce the complexity of the algorithms used. Furthermore, companies should be transparent about any cases of discrimination and the number of claims by applicants, which could be reported in the form of a transparency report.

The bottom line is this: all the aspects outlined above are mutually supportive, and we must develop an integrated approach to ensure the ethical use of AI recruiting tools.

## Conclusion

Our article demonstrates that a complete ethical condemnation of AI is not justified from a human rights perspective because AI recruiting does not inherently conflict with human rights. In our normative background section, we first outline which human rights are relevant in the recruiting context. Furthermore, we illustrate how AI’s specific properties challenge the fulfillment of these rights and derive ethical implications for AI recruiting, which are manifested in the following principles: validity, autonomy, nondiscrimination, privacy, and transparency. In our subsequent normative analysis, we analyze whether AI recruiting inherently conflicts with these derived principles and argue that AI recruiting should not be considered unethical per se. We posit that whether AI recruiting conflicts with the examined ethical principles heavily depends on the conditions under which AI recruiting tools are used. We derive concrete implications for and responsibilities of organizations to enforce and realize human rights standards in the context of AI recruiting. However, we further argue that a realistic approach is needed, whereby human rights and ethical principles are not interpreted in their strictest forms. Rather, an actionable approach must address human rights in recruiting while leaving sufficient room for new technological developments, which inevitably will lead to adjusted processes, changes in recruiters’ responsibilities, and new requirements for applicants.

With our theoretical work, in which we normatively assess the topic of AI recruiting, we aim to bridge the gap between business ethics and AI recruiting applications in practice. Furthermore, we aim to provide organizations with guidance on deploying AI in the selection process by outlining the ethical implications for recruiting that are related to human rights, as well as organizations’ related responsibilities.

## Data Availability

Not applicable.

## Footnotes

1 The UN Guiding Principles refer here explicitly to the International Bill of Rights, which includes the Universal Declaration of Human Rights, the International Covenant on Civil and Political Rights, and the International Covenant on Economic, Social and Cultural Rights, and to the principles established in the Declaration of Fundamental Principles and Rights at Work.

2 Meaning the Universal Declaration of Human Rights, the International Covenant on Civil and Political Rights, and the International Covenant on Economic, Social and Cultural Rights.

3 In addition to the general debate on AI ethics, specific human rights implications of AI have been studied quite recently [47–49]. Specifically, the UN Report on Artificial Intelligence [50] has studied the human rights implications of AI and made references to the impact of AI on freedom of speech and on discrimination.

4 Intersectional discrimination refers to discrimination that occurs on the basis of multiple personal grounds or characteristics that simultaneously interact with each other in a way that makes them inseparable [80].

## References

[1] Köchling A, Wehner MC (2020). Discriminated by an algorithm: a systematic review of discrimination and fairness by algorithmic decision-making in the context of HR recruitment and HR development. Bus. Res..

[2] Daugherty PR, Wilson HJ (2018). Human + Machine: Reimagining Work in the Age of AI.

[3] Mujtaba, D.F., Mahapatra, N.R.: Ethical considerations in AI-based recruitment. In: Cunningham, M., Cunningham, P. (eds.) 2019 IEEE International Symposium on Technology in Society (ISTAS) Proceedings. IEEE International Symposium on Technology in Society, Medford, MA, USA, November 15–16, pp. 1–7. IEEE (2019). 10.1109/Istas48451.2019.8937920

[4] Giermindl LM, Strich F, Christ O, Leicht-Deobald U, Redzepi A (2021). The dark sides of people analytics: reviewing the perils for organisations and employees. Eur. J. Inf. Syst..

[5] Precht RD (2020). Künstliche Intelligenz Und Der Sinn Des Lebens.

[6] Chamorro-Premuzic, T.: Will AI reduce gender bias in hiring? Harv. Bus. Rev. **June 10** (2019)

[7] Polli, F.: Using AI to eliminate bias from hiring. Harv. Bus. Rev. **October 29** (2019)

[8] Donaldson, T., Dunfee, T.W.: When ethics travel: the promise and peril of global business ethics. Calif. Manag. Rev. **41** (1999)

[9] Ruggie JG (2007). Business and human rights: the evolving international Agenda. Am. J. Int. Law.

[10] Santoro MA (2015). Business and human rights in historical perspective. J. Hum. Rights.

[11] Yam J, Skorburg JA (2021). From human resources to human rights: impact assessments for hiring algorithms. Ethics Inf. Technol..

[12] Latonero, M.: Governing artificial intelligence. Upholding human rights & dignity. Data & Society, pp. 1–37 (2018)

[13] Enderle G (2021). Corporate Responsibility for Wealth Creation and Human Rights.

[14] Hagendorff T (2020). The ethics of AI ethics: an evaluation of guidelines. Minds Mach..

[15] High-level expert group on artificial intelligence: ethics guidelines for trustworthy AI. https://ec.europa.eu/digital-single-market/en/news/ethics-guidelines-trustworthy-ai (2019). Accessed 13 Aug 2020

[16] Tolmeijer S, Kneer M, Sarasua C, Christen M, Bernstein A (2020). Implementations in machine ethics: a survey. ACM Comput. Surv..

[17] Floridi L, Cowls J, Beltrametti M, Chatila R, Chazerand P, Dignum V, Lütge C, Madelin R, Pagallo U, Rossi F, Schafer B, Valcke P, Vayena E (2018). AI4people—an ethical framework for a good AI society: opportunities, risks, principles, and recommendations. Minds Mach..

[18] Polli F, Dolphin J, Kassir S (2019). On the basis of brains: how neuroscience and AI advance ethical hiring. Workforce Solut. Rev..

[19] Vasconcelos, M., Cardonha, C., Gonçalves, B.: Modeling epistemological principles for bias mitigation in AI systems: an illustration in hiring decisions. In: Furman, J., Marchant, G., Price, H., Rossi, F. (eds.) Proceedings of the 2018 AAAI/ACM Conference on AI, Ethics, and Society. AAAI/ACM Conference on AI, Ethics, and Society, New Orleans, LA, USA, February 2–3, pp. 323–329. ACM, New York, NY, USA (2018). 10.1145/3278721.3278751

[20] Hunkenschroer AL, Lütge C (2021). How to improve fairness perceptions of AI in hiring: the crucial role of positioning and sensitization. AI Ethics J..

[21] Lee MK (2018). Understanding perception of algorithmic decisions: fairness, trust, and emotion in response to algorithmic management. Big Data Soc..

[22] Simbeck K (2019). HR analytics and ethics. IBM J. Res. Dev..

[23] Hunkenschroer AL, Luetge C (2022). Ethics of AI-enabled recruiting and selection: a review and research agenda. J. Bus. Ethics.

[24] Kaplan A, Haenlein M (2019). Siri, Siri, in my hand: who’s the fairest in the land? On the interpretations, illustrations, and implications of artificial intelligence. Bus. Horiz..

[25] Black JS, Van Esch P (2020). AI-enabled recruiting: what is it and how should a manager use it?. Bus. Horiz..

[26] Bogen, M.: All the ways hiring algorithms can introduce bias. Harv. Bus. Rev. **May 6** (2019)

[27] Rąb-Kettler K, Lehnervp B (2019). Recruitment in the times of machine learning. Manag. Syst. Prod. Eng..

[28] Köchling A, Riazy S, Wehner MC, Simbeck K (2020). Highly accurate, but still discriminatory. A fairness evaluation of algorithmic video analysis in the recruitment context. Bus. Inf. Syst. Eng..

[29] Tambe P, Cappelli P, Yakubovich V (2019). Artificial intelligence in human resources management: challenges and a path forward. Calif. Manag. Rev..

[30] Van Esch P, Black JS (2019). Factors that influence new generation candidates to engage with and complete digital, AI-enabled recruiting. Bus. Horiz..

[31] Santoro MA (2000). Profits and Principles: Global Capitalism and Human Rights in China.

[32] Friedman, M.: The social responsibility of business is to increase its profits. The New York Times, 13 September 1970

[33] Donaldson T, Dunfee TW (1995). Integrative social contracts theory: a communitarian conception of economic ethics. Econ. Philos..

[34] Wettstein F (2015). Normativity, ethics, and the unguiding principles on business and human rights: a critical assessment. J. Hum. Rights.

[35] United Nations: Guiding principles on business and human rights. https://www.ohchr.org/documents/publications/guidingprinciplesbusinesshr_en.pdf (2011). Accessed 10 Mar 2022

[36] Kriebitz A, Lütge C (2020). Artificial intelligence and human rights: a business ethical assessment. Bus. Hum. Rights J..

[37] Baumhart JT (1992). The employer's right to read employee e-mail: protecting property or personal prying?. Labor Lawyer.

[38] Cavico FJ (1993). Invasion of privacy in the private employment sector: tortious and ethical aspects. Hous. Law Rev..

[39] Sandeen SK, Mylly UM (2019). Trade secrets and the right to information: a comparative analysis of EU and us approaches to freedom of expression and whistleblowing. N. C. J. Law Technol..

[40] Allgemeines Gleichbehandlungsgesetz (Agg): General act on equal treatment. https://www.antidiskriminierungsstelle.de/en/about-discrimination/order-and-law/general-equal-treatment-act/general-equal-treatment-act-node (2006). Accessed 10 Mar 2022

[41] Alder GS, Gilbert J (2006). Achieving ethics and fairness in hiring: going beyond the law. J. Bus. Ethics.

[42] Mittelstadt BD, Allo P, Taddeo M, Wachter S, Floridi L (2016). The ethics of algorithms: mapping the debate. Big Data Soc..

[43] Van Otterlo M, Hildebrandt M, Vries KD (2013). A machine learning view on profiling. Privacy, Due Process and the Computational Turn-Philosophers of Law Meet Philosophers of Technology.

[44] Pasquale F (2015). The Black Box Society.

[45] Diakopoulos N (2015). Algorithmic accountability: journalistic investigation of computational power structures. Digit. J..

[46] University of Montreal: Montreal declaration for a responsible development of artificial intelligence. https://www.montrealdeclaration-responsibleai.com/the-declaration (2018)

[47] Fjeld J, Achten N, Hilligoss H, Nagy AC, Srikumar M (2020). Principled Artificial Intelligence: Mapping Consensus in Ethical and Rights-Based Approaches to Principles for AI.

[48] Humble KP, Altun D (2020). Artificial intelligence and the threat to human rights. J. Internet Law.

[49] ZuiderveenBorgesius FJ (2020). Strengthening legal protection against discrimination by algorithms and artificial intelligence. Int. J. Hum. Rights.

[50] Kaye, D.: Promotion and protection of the right to freedom of opinion and expression, A/73/348 (2018)

[51] Yarger L, Cobb Payton F, Neupane B (2020). Algorithmic equity in the hiring of underrepresented it job candidates. OIR.

[52] Cragg W, Sójka J, Wempe J (2000). Human rights and business ethics: fashioning a new social contract. Business Challenging Business Ethics: New Instruments for Coping with Diversity in International Business.

[53] Macaskill W (2016). Doing Good Better: How Effective Altruism can Help You Help Others, Do Work that Matters, and Make Smarter Choices about Giving Back.

[54] Acikgoz Y, Davison KH, Compagnone M, Laske M (2020). Justice perceptions of artificial intelligence in selection. Int. J. Select. Assess..

[55] Chamorro-Premuzic, T., Akhtar, R.: Should companies use AI to assess job candidates? Harv. Bus. Rev. **May 17** (2019)

[56] Florentine, S.: How artificial intelligence can eliminate bias in hiring. CIO, 22 December 2016. https://www.cio.com/article/3152798/how-artificial-intelligence-can-eliminate-bias-in-hiring.html. Accessed 20 Jul 2020

[57] Faraj S, Pachidi S, Sayegh K (2018). Working and organizing in the age of the learning algorithm. Inf. Org..

[58] Gal, U., Jensen, T.B., Stein, M.K.: People analytics in the age of big data: an agenda for is research. In: International Conference on Information Systems, Seoul, South Korea (2017)

[59] Delandshere G, Petrosky AR (1998). Assessment of complex performances: limitations of key measurement assumptions. Educ. Res..

[60] Govaerts M, Van Der Vleuten CPM (2013). Validity in work-based assessment: expanding our horizons. Med. Educ..

[61] Lantolf JP, Frawley W (1988). Proficiency. Understanding the construct. Stud Sec Lang Acq.

[62] Chamorro-Premuzic, T., Polli, F., Dattner, B.: Building ethical AI for talent management. Harv. Bus. Rev. **November 21** (2019)

[63] Raghavan, M., Barocas, S., Kleinberg, J., Levy, K.: mitigating bias in algorithmic hiring: evaluating claims and practices. In: Proceedings of the FAT* '20: Conference on Fairness, Accountability, and Transparency. Conference on Fairness, Accountability, and Transparency, Barcelona, Spain, January 27–30. Association for Computing Machinery, New York, USA (2020)

[64] Dattner, B., Chamorro-Premuzic, T., Buchband, R., Schettler, L.: The legal and ethical implications of using AI in hiring. Harv. Bus. Rev. **April 25** (2019)

[65] Chamorro-Premuzic T, Akhtar R, Winsborough D, Sherman RA (2017). The datafication of talent: how technology is advancing the science of human potential at work. Curr. Opin. Behav. Sci..

[66] Appelbaum D, Kogan A, Vasarhelyi M, Yan Z (2017). Impact of business analytics and enterprise systems on managerial accounting. Int. J. Account. Inf. Syst..

[67] Durand R (2003). Predicting a firm’s forecasting ability: the roles of organizational illusion of control and organizational attention. Strateg. Manag. J..

[68] Chamorro-Premuzic T, Winsborough D, Sherman, Ryne A, Hogan R (2016). New talent signals: shiny new objects or a brave new world?. Ind. Organ. Psychol..

[69] Kahnemann D (2011). Thinking, Fast and Slow.

[70] Bartneck C, Lütge C, Wagner A, Welsh S (2021). An Introduction to Ethics in Robotics and AI.

[71] Lawless, W.F., Sofge, D.A.: Evaluations: autonomy and artificial intelligence: a threat or savior? In: Autonomy and Artificial Intelligence: A Threat or Savior? Springer, Cham, pp. 295–316 (2017)

[72] Chesterman S (2020). Artificial intelligence and the problem of autonomy. Notre Dame J. Emerg. Technol..

[73] Curchod C, Patriotta G, Cohen L, Neysen N (2020). Working for an algorithm: power asymmetries and agency in online work settings. Admin. Sci. Q..

[74] Kellogg KC, Valentine MA, Christin A (2020). Algorithms at work: the new contested terrain of control. Acad. Manag. Ann..

[75] Lin Y-T, Hung T-W, Huang LT-L (2020). Engineering equity: how AI can help reduce the harm of implicit bias. Philos. Technol..

[76] Van Den Hoven, J., Manders-Huits, N.: the person as risk, the person at risk. In: ETHICOMP: Living, Working And Learning Beyond Technology, Mantua, Italy, September 24–26, pp. 408–414. (2008)

[77] Wachter S, Mittelstadt B, Russell C (2020). Why fairness cannot be automated: bridging the gap between EU non-discrimination law and AI. Comput. Law Secur. Rev..

[78] Newman DT, Fast NJ, Harmon DJ (2020). When eliminating bias isn’t fair: algorithmic reductionism and procedural justice in human resource decisions. Organ. Behav. Hum. Decis. Process..

[79] Mann M, Matzner T (2019). Challenging algorithmic profiling: the limits of data protection and anti-discrimination in responding to emergent discrimination. Big Data Soc..

[80] European Union Agency For Fundamental Rights: homophobia and discrimination on grounds of sexual orientation and gender identity in the EU member states. Part II—the social situation. https://fra.europa.eu/sites/default/files/fra_uploads/397-fra_hdgso_report_part2_en.pdf (2009)

[81] Persson A, Lehmann A, Whitehouse D, Fischer-Hübner S, Fritsch L, Raab C (2016). Implicit bias in predictive data profiling within recruitments. Privacy and Identity Management. Facing Up to Next Steps.

[82] Mann, G., O’neil, C.: hiring algorithms are not neutral. Harv. Bus. Rev. **December 9** (2016)

[83] Fernández-Martínez C, Fernández A (2020). AI and recruiting software: ethical and legal implications. Paladyn J. Behav. Robot..

[84] Bîgu, D., Cernea, M.-V.: Algorithmic bias in current hiring practices: an ethical examination. In: 13th International Management Conference (IMC) on Management Strategies for High Performance. 13th International Management Conference (IMC) on Management Strategies for High Performance, Bucharest, Romania, October 31–November 1 (2019)

[85] Scott-Parker, S.: AI powered disability discrimination. AI ethics: global perspectives, 2 June 2021. https://www.youtube.com/watch?v=1n5v_w9saos. Accessed 9 Aug 2021

[86] United Nations: Universal Declaration of Human Rights. https://www.un.org/en/about-us/universal-declaration-of-human-rights (1948). Accessed 10 Mar 2022

[87] Bverfg: order of the first senate 13 June 2007, Paras. 1–151. http://www.bverfg.de/e/rs20070613_1bvr178305en.html (2007). Accessed 10 Mar 2022

[88] Sánchez-Monedero, J., Dencik, L., Edwards, L.: what does it mean to ‘solve’ the problem of discrimination in hiring? Social, technical and legal perspectives from the UK on automated hiring systems. In: Proceedings of the FAT*'20: Conference on Fairness, Accountability, and Transparency. Conference on Fairness, Accountability, and Transparency, Barcelona, Spain, January 27–30, pp. 458–468. Association For Computing Machinery, New York, USA (2020). 10.1145/3351095.3372849

[89] Oswald FL, Behrend TS, Putka DJ, Sinar E (2020). Big data in industrial-organizational psychology and human resource management: forward progress for organizational research and practice. Annu. Rev. Organ. Psychol. Organ. Behav..

[90] Jayaratne M, Jayatilleke B (2020). Predicting personality using answers to open-ended interview questions. IEEE Access.

[91] Pena, A., Serna, I., Morales, A., Fierrez, J.: Bias in multimodal AI: testbed for fair automatic recruitment. In: 2020 IEEE/CVF Conference on Computer Vision and Pattern Recognition Workshops. 2020 IEEE/CVF Conference on Computer Vision and Pattern Recognition Workshops (CVPRW), Seattle, WA, USA, June 14–19, pp. 129–137. IEEE (2020). 10.1109/Cvprw50498.2020.00022

[92] Kim TW, Routledge BR (2021). Why a right to an explanation of algorithmic decision-making should exist: a trust-based approach. Bus Ethics Q.

[93] Desai DR, Kroll JA (2017). Trust but verify: a guide to algorithms and the law. Harv. J. Law Technol..

[94] Ananny M, Crawford K (2018). Seeing without knowing: limitations of the transparency ideal and its application to algorithmic accountability. New Media Soc..

[95] Mantelero A (2018). AI and big data: a blueprint for a human rights, social and ethical impact assessment. Comput. Law Secur. Rev..

[96] Aizenberg E, Van Den Hoven J (2020). Designing for human rights in AI. Big Data Soc..

